# Novel Loss-of-Function *SYCP2* Variants in Infertile Males Upgrade the Gene–Disease Clinical Validity Classification for *SYCP2* and Male Infertility to Strong

**DOI:** 10.3390/genes15081092

**Published:** 2024-08-19

**Authors:** Jinli Li, Samantha L.P. Schilit, Shanshan Liang, Ningxin Qin, Xiaoming Teng, Junyu Zhang

**Affiliations:** 1 Reproductive Medicine Center, Shanghai First Maternity and Infant Hospital, School of Medicine, Tongji University, 2699 West Gaoke Road, Shanghai 201204, China; lijinli@51mch.com (J.L.); liangshanshan@51mch.com (S.L.); ningxinqin@51mch.com (N.Q.); 2 Harvard Medical School, Boston, MA 02115, USA; samanthaschilit@gmail.com

**Keywords:** *SYCP2*, autosomal dominant, gene–disease association, male infertility, oligoasthenozoospermia

## Abstract

Male infertility affects approximately 7% of the male population, and about 15% of these cases are predicted to have a genetic etiology. One gene implicated in autosomal dominant male infertility, *SYCP2*, encodes a protein critical for the synapsis of homologous chromosomes during meiosis I, resulting in impaired spermatogenesis. However, the clinical validity of the gene–disease pair was previously categorized as on the border of limited and moderate due to few reported cases. This study investigates the genetic cause of infertility for three unrelated Chinese patients with oligoasthenozoospermia. Whole exome sequencing (WES) and subsequent Sanger sequencing revealed novel heterozygous loss-of-function (LOF) variants in *SYCP2* (c.89dup, c.946_947del, and c.4378_4379del). These cases, combined with the previously reported cases, provide strong genetic evidence supporting an autosomal dominant inheritance pattern. The experimental evidence also demonstrates a critical role for *SYCP2* in spermatogenesis. Collectively, this updated assessment of the genetic and experimental evidence upgrades the gene–disease association strength of *SYCP2* and autosomal dominant male infertility from on the border of limited and moderate to strong. The reclassification improves *SYCP2* variant interpretation and qualifies it for the inclusion on diagnostic male infertility gene panels and prioritization in whole exome or genome studies for related phenotypes. These findings therefore improve the clinical interpretation of *SYCP2* LOF variants.

## 1. Introduction

Male infertility is a complex and multifactorial condition that affects approximately 7% of men. Genetic factors contribute to at least 15% of the male infertility cases and can cause issues in four main categories: hypothalamic–pituitary axis disturbances, ductal obstruction or dysfunction, spermatogenic qualitative defects, and spermatogenic quantitative defects [1]. Understanding the genetic etiology of male infertility may inform the genetic counseling and therapeutic interventions available to the patient, such as hormonal therapies, extraction of sperm from the epididymis or testicle, intracytoplasmic sperm injection (ICSI) and in vitro fertilization (IVF), or seeking donor sperm or adoption [2,3]. Therefore, identifying the genetic variants implicated in human male infertility can provide meaningful and actionable information to the patient.

While the identification of novel candidate genes in infertile males has increased rapidly since the implementation of next-generation sequencing, many genes have not yet accumulated sufficient evidence to be confidently implicated in male infertility [4]. The accurate interpretation of genomic variants is critical for understanding the clinical significance of a genetic test result, yet the clinical interpretation for variants in a gene cannot be provided unless the gene–disease association has been established [5]. Therefore, a clear understanding of the clinical validity of gene–disease relationships is a fundamental prerequisite for accurate variant interpretation in genes thought to be important for male fertility. The Clinical Genome Resource (ClinGen) has developed a semiquantitative points-based framework for assigning clinical validity classifications to gene–disease relationships [6,7]. This framework involves curating the published literature to evaluate the strength of the genetic and experimental evidence. Based on this evaluation, the clinical validity can be categorized as definitive (12–18 points and replicated over time), strong (12–18 points), moderate (7–11 points), limited (0.1–6 points), no known disease relationship (0 points), disputed (with contradictory evidence), or refuted (with contradictory evidence that outweighs the supportive evidence) per version 9 of the ClinGen Gene–Disease Validity Standard Operating Procedure (SOP) [6]. Gene–disease associations may be reclassified as additional evidence accumulates in the literature. Updated classifications assist clinical laboratories in deciding which genes to add to disease-targeted panels and how to prioritize the variants from whole exome sequencing (WES) or whole genome sequencing (WGS) data. 

*SYCP2* (cytogenetic location: 20q13.33) is a novel candidate gene that, despite substantial experimental evidence, has only achieved a classification of borderline limited to moderate when evaluated for an association with autosomal dominant male infertility [8]. In its last comprehensive assessment, three frameshift variants in *SYCP2* were identified in men with cryptozoospermia or azoospermia, suggesting that heterozygous loss-of-function (LOF) variants in *SYCP2* might be responsible for the low sperm count and subsequent infertility [8]. There is substantial experimental evidence supporting a role for *SYCP2* in male infertility. *SYCP2* encodes synaptonemal complex protein 2, an axial element in the proteinaceous synaptonemal complex (SC) [9,10]. SC assembly contributes to the pairing and segregation of homologous chromosomes during meiosis I [11]. *SYCP2* is important for spermatogenesis as mice lacking the coiled-coil domain of *Sycp2* exhibit impaired homologous chromosome synapsis, leading to spermatocyte apoptosis and male-specific infertility [12].

In this study, we report three unrelated Chinese patients with oligoasthenozoospermia and heterozygous *SYCP2* frameshift variants. The inclusion of these cases, along with the additional evidence from the literature, upgrades the strength of the gene–disease association for *SYCP2* and male infertility from on the border of limited and moderate to strong. The upgraded classification provides sufficient clinical validity to improve *SYCP2* variant interpretation and to qualify it for inclusion on diagnostic male infertility gene panels and prioritization in WES or WGS studies for related phenotypes.

## 2. Patients and Methods

### 2.1. Patients and Ethics Statement

A total of 537 participants with male infertility were recruited from the Reproductive Genetics Clinic at Shanghai First Maternity and Infant Hospital. Following WES of the entire cohort, LOF variants in *SYCP2* were identified in three probands, who were investigated further for this study. All three had been diagnosed with oligoasthenozoospermia in accordance with the fifth edition of the WHO Laboratory Manual for the Examination and Processing of Human Semen (oligozoospermia [low sperm count, below 15 million/mL] and asthenozoospermia [progressive motility below 32%]) [13]. This study was approved by the medical ethics committee of Shanghai First Maternity and Infant Hospital (XJ2407), and all participants provided written informed consent.

### 2.2. Whole Exome Sequencing (WES), Variant Calling, and Validation of Sequence Variants

Participant genomic DNA was isolated from peripheral blood lymphocytes using QIAamp DNA Blood Kits (Qiagen, Hilden, Germany). Library preparation and exome enrichment were performed using SureSelect Human All Exon V6 (Agilent Technologies, Santa Clara, CA, USA). The prepared libraries were then sequenced with the Illumina NovaSeq^®^ 6000 system (Illumina, San Diego, CA, USA). The original sequencing data were mapped to the human genome assembly GRCh37/hg19 using Burrows–Wheeler Aligner (BWA) [14]. After alignment, Picard tools removed PCR duplicates and evaluated the quality of the sequencing data. Variant calling and annotation were performed using the Genome Analysis Toolkit (GATK) [15]. The obtained variants were filtered and prioritized using TGex (https://fa.shanyint.com/, accessed on 25 May 2024) [16]. Genes with a definitive, strong, moderate, or limited strength association to male infertility were prioritized. The gene variants detected by WES were verified by Sanger sequencing. Amplification and subsequent Sanger sequencing were performed using the following primers: Family 1, *SYCP2*-c.89dup-F = GGTTTTGATACAGTATGTGCCATT and *SYCP2*-c.89dup-R = AATTATGACTGAGCTTGCCCA; Family 2, *SYCP2*-c.946_947del-F = CTTGCTTTAGCCATGAATATAACAT and *SYCP2*-c.946_947del-R = ATTTATAATTAGCTTGGTAGGTTGCT; Family 3: *SYCP2*-c.4378_4379del-F = CTTTGATAATGAGAAATCCTGAGAGA and *SYCP2*-c.4378_4379del-R = CAGAAGACACTTTTAGCCAATGAA.

### 2.3. Ovarian Stimulation, Fertilization, Embryo Culture, and Embryo Evaluation

Partners of affected subjects underwent ovarian stimulation using a GnRH agonist/antagonist protocol and oocyte retrieval as previously described [17]. Approximately two hours after oocyte retrieval, fertilization was performed via ICSI using sperm obtained from semen samples. The embryos were cultured in G-IVF and G-1 media (Vitrolife, Goteborg, Sweden) in a benchtop incubator (MIRI^®^ Multiroom Incubator, ESCO, Singapore). The incubator maintained a 6% CO_2_ concentration and 37 °C temperature.

Embryos were graded according to the standardized grading system established by the Society for Assisted Reproductive Technique (SART) committee [18].

### 2.4. Literature Review and Gene–Disease Clinical Validity Curation

A thorough literature search was performed using PubMed and Google Scholar to identify publications describing the relationship between *SYCP2* and male infertility in both humans and animal models. Only manuscripts or abstracts in English were evaluated. Cases from the literature with deleterious *SYCP2* variants and a phenotype of male infertility due to a low sperm count (i.e., cryptozoospermia, oligoasthenozoospermia, or azoospermia) were reviewed. 

The strength of the gene–disease association between *SYCP2* and autosomal dominant male infertility was curated according to the ClinGen Gene–Disease Validity SOP, version 9 [6].

## 3. Results 

### 3.1. Clinical Description of Three Chinese Individuals with Oligoasthenozoospermia

**Figure 1:**
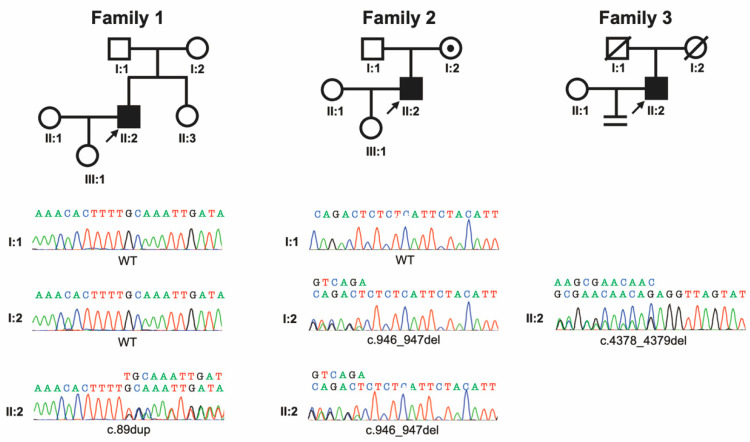
Pedigrees and genotypes for subjects in this study. The three probands with oligoasthenozoospermia and their families are represented. (**Top**): Pedigrees are depicted using standardized pedigree nomenclature. (**Bottom**): Sanger sequencing chromatogram traces are provided for tested individuals.

Patient 1, a 30-year-old male (Family 1, II:2), was diagnosed with male infertility resulting from oligoasthenozoospermia, characterized by a semen analysis with only 3–4 forward motile sperm within a high-power field of view and a semen volume of 6.0 mL. An endocrine evaluation identified no abnormal values (follicle-stimulating hormone (FSH) = 2.11 IU/L [reference range 1–7 IU/L]; luteinizing hormone (LH) = 2.41 IU/L [reference range 1.5–9.3 IU/L]; and testosterone = 5.86 nmol/L [reference range 1.23–8.14 nmol/L]). An evaluation for female factor infertility and the karyotypes for both partners were unremarkable. As an intervention for male infertility, IVF was performed using sperm retrieved from a semen sample. A total of fifteen cumulus–oocyte complexes were retrieved after ovarian stimulation, resulting in ten metaphase II (MII) oocytes, including eight that fertilized and cleaved normally after ICSI. Following the transfer of a single good-quality day-three embryo (cell number: 8; fragmentation: <10%; inner cell mass: perfectly symmetrical) [18,19], the couple achieved the birth of a healthy female weighing 3.00 kg at 39 weeks of gestation by caesarian section (III:1) (Figure 1).

Patient 2, a 35-year-old male (Family 2, II:2), was diagnosed with oligoasthenozoospermia. A semen analysis revealed a semen volume of 3.0 mL, a sperm concentration of 4.3 million per milliliter, low progressive motility (4.9%), and a normal morphology rate of <1%. An endocrine evaluation was unremarkable (FSH = 3.07 IU/L [reference range 1–7 IU/L]; LH = 2.78 IU/L [reference range 1.5–9.3 IU/L]; and testosterone = 6.08 nmol/L [reference range 1.23–8.14 nmol/L]). No fertility concerns were identified for patient 2’s reproductive partner, and the karyotypes for both individuals were normal. The couple pursued IVF as a treatment for male infertility. A total of three cumulus–oocyte complexes were retrieved after ovarian stimulation, including two metaphase II (MII) oocytes, of which one fertilized with normal cleavage after ICSI using the sperm retrieved from a semen sample. Following the transfer of the single good-quality day-three embryo (cell number: 8; fragmentation: <10%; inner cell mass: perfectly symmetrical) [18,19], the couple gave birth to a healthy female weighing 3.60 kg at 40 weeks of gestation by caesarian section (III:1) (Figure 1).

Patient 3, a 47-year-old male (Family 3, II:2), was diagnosed with severe oligoasthenozoospermia. He had suffered from primary male infertility for years, and a semen analysis revealed only one immobile sperm within a high-power field of view with a semen volume of 2.1 mL. An endocrine evaluation was normal (FSH = 2.05 IU/L [reference range 1–7 IU/L]; LH = 2.08 IU/L [reference range 1.5–9.3 IU/L]; and testosterone = 5.56 nmol/L [reference range 1.23–8.14 nmol/L]). An evaluation for female factor infertility and the karyotypes for both partners were unremarkable. Despite multiple attempts at ICSI and in spite of successful fertilizations, the couple was unable to obtain viable embryos. The patient eventually discontinued the treatment (Figure 1).

### 3.2. Genetic Analysis Identifies Novel Heterozygous Loss-of-Function (LOF) SYCP2 Variants in the Patients

To investigate the genetic factors contributing to the infertility of these patients, WES and subsequent validation through Sanger sequencing were performed (Table 1). In patient 1, this investigation resulted in the identification of a heterozygous variant in exon 4 of *SYCP2*, NM_014258.4:c.89dup, causing p.(Leu30Phefs*4). Sanger sequencing of the parental samples with confirmed parentage revealed that neither of his parents carried this variant, suggesting that it arose *de novo* in the patient. Patient 2 was found to have a heterozygous variant in exon 14 of *SYCP2*, NM_014258.4:c.946_947del, causing p.(Ser316Ilefs*7). Sanger sequencing of the parental samples demonstrated maternal inheritance. In patient 3, a heterozygous variant in exon 42 of *SYCP2* was found, NM_014258.4:c.4378_4379del, causing p.(Arg1460Alafs*10). As both of his parents are deceased, the origin of his variant remains unknown. All three variants are predicted to cause loss of function through nonsense-mediated decay given that they result in premature termination codons that reside more than 50 bp upstream of the last exon–exon junction [20]. In addition, all three are absent from any publications and rare in the Genome Aggregation Database (gnomAD, v4.1.0), with zero alleles for NM_014258.4:c.89dup and only one allele for both NM_014258.4:c.946_947del (1/1,585,786 alleles = allele frequency of 6.306 × 10^−7^) and NM_014258.4:c.4378_4379del (1/1,412,392 alleles = allele frequency of 7.080 × 10^−7^) (http://gnomad.broadinstitute.org) [21].

### 3.3. The Addition of Three Cases Upgrades the Strength of the Gene–Disease Relationship between SYCP2 and Autosomal Dominant Male Infertility to Strong

The three cases identified in this study prompted a re-evaluation of the gene–disease relationship between *SYCP2* and male infertility, which involved an investigation of the strength of both the genetic and experimental evidence. When combined with the previous cases identified in the literature, six individuals with male infertility resulting from a low sperm count have been found to have heterozygous LOF variants in *SYCP2* (Table 1) [8]. As would be expected for a variant that decreases reproductive fitness, all these variants are absent from or rare in gnomAD [21]. Additional supportive evidence for pathogenicity comes from patient 1 with the c.89dup variant that occurred *de novo* (this study). By applying these cases using the ClinGen Gene–Disease Validity SOP, the cumulative genetic evidence scored 9.5 out of 12 total allowable points (Table 2) [6]. 

The correlation between *SYPC2* and male infertility is also supported strongly by experimental evidence (Table 3). *SYCP2* encodes synaptonemal complex protein 2, an axial element in the SC, which plays a key role in meiosis I during spermatogenesis [9,10,22]. *SYCP2* exhibits predominant expression in testis tissue, where spermatogenesis occurs [9,23]. Studies on *Saccharomyces cerevisiae* suggest that *SYCP2* is critical for SC formation as mutant *red1*, the yeast functional homolog of *SYCP2,* fails to assemble the SC during meiotic prophase [24]. A loss of function results in impaired spermatogenesis and infertility, as has been observed in models including *sycp2*^−/−^ zebrafish and homozygous coiled-coil domain-deficient *Sycp2* mice, as well as in humans [8,12,25]. Additionally, the coiled-coil domain of murine SYCP2 interacts with SYCP3 and TEX11, which are themselves implicated in human male infertility [12,26,27,28]. These data highlight a critical role for *SYCP2* in male reproductive biology, with a cumulative evidence score of the maximum 6 out of 6 allowable points per the ClinGen Gene–Disease Validity SOP (Table 3) [6].

By combining the cumulative points from the genetic (9.5 points) and experimental (6 points) evidence, this gene–disease curation achieves a total score of 15.5 out of 18 allowable points. Based upon the scoring by the ClinGen Gene–Disease Validity SOP, the strength of the gene–disease association between *SYCP2* and autosomal dominant male infertility is classified as strong (12–18 points required) [6]. 

## 4. Discussion

Despite substantial experimental evidence, the clinical validity of the gene–disease association between *SYCP2* and male infertility had been classified as borderline limited to moderate previously due to insufficient case evidence [8]. This study doubles the number of cases of infertile men with heterozygous LOF variants in *SYCP2,* resulting in an upgraded ClinGen gene–disease clinical validity categorization to strong.

The gene–disease validity classification informs gene panel inclusion and variant classification. For clinical diagnostic panels, gene–disease associations with evidence classified as definitive, strong, or moderate may be included to maximize sensitivity [29]. However, for predictive or screening purposes, it is recommended to only include genes classified as having definitive or strong gene–disease associations in order to minimize false positives [30]. The reclassification of the strength of *SYCP2* and male infertility gene–disease association from on the border of limited and moderate to strong provides justification for now incorporating *SYCP2* into male infertility gene panels. In addition, the strength of the gene–disease association has a direct impact on variant classification. The American College of Medical Genetics and Genomics (ACMG) has recommended that variants within genes categorized as limited should not be classified at a higher level than variants of uncertain significance (VUS). In contrast, for genes with a disease association categorized as definitive or strong, variants can be classified as high as pathogenic [31]. Thus, this study enables an upgrade of *SYCP2* LOF variants from VUS to more deleterious classifications as appropriate. 

Of note, this study focuses on the relationship between the heterozygous LOF variants in *SYCP2* and male infertility, thereby specifying the proposed mechanism of pathogenicity and mode of inheritance. Three additional cases were identified in the literature but not applied to this curation because they did not meet the criteria for this gene–disease pair. First, one individual with non-obstructive azoospermia resulting from Sertoli cell-only syndrome was found to have a heterozygous intronic variant (c.298-3dup, p.?) in *SYCP2* [32]. This variant duplicates a base in a homopolymer region immediately adjacent to the splice site in a U12-type intron. While this variant might impact splicing [33], there is no evidence of gene impact, which would enable its inclusion as genetic evidence in the curation. Second, a case with severe oligozoospermia was found to have 46,XY,t(20;22)(q13.3;q11.2) that resulted in *SYCP2* overexpression [8]. Despite the functional studies that support a model in which overexpression leads to the aberrant assembly of the SC, which suggests a similar LOF mechanism of pathogenicity [8], a single case is not sufficient genetic evidence to classify the association between *SYCP2* overexpression and male infertility beyond limited at this time. Similarly, a third case was reported with non-obstructive azoospermia and a homozygous variant in *SYCP2*, c.2689_2690insT, causing p.(Ala897Valfs*5) [34]. This proband was conceived naturally by heterozygous parents, suggesting an autosomal recessive mode of inheritance [34]. Of note, no information was reported regarding the sperm count or quality from the proband’s father, although natural conception implies sufficient fertility. This single case with an autosomal recessive mode of inheritance is once again insufficient genetic evidence to classify the association between *SYCP2* and autosomal recessive male infertility beyond limited. 

These cases highlight a couple of observations about the gene–disease association between *SYCP2* and male infertility and opportunities for future study. First, the variant spectrum of *SYCP2*-mediated male infertility has not yet been fully elucidated. Second, while variable expressivity regarding the extent of spermatogenesis impairment is a known feature of *SYCP2*-mediated infertility [8], the penetrance of the disorder is unknown due to the ascertainment of affected cases. Indeed, the presumed unaffected father of the azoospermic proband with a homozygous LOF variant in *SYCP2* suggests incomplete penetrance in the heterozygous state [34]. However, the extreme intolerance to loss of function according to constraint analyses of population datasets might lead to the hypothesis of a more penetrant condition given the presumed impact on reproductive fitness [21]. For this reason, while identifying the LOF variants in *SYCP2* would be helpful in a diagnostic setting for male infertility, its utility in screening and predicting disease has yet to be established. We encourage reports of additional cases that can help to address these critical questions. As demonstrated by the successful use of assisted reproductive technologies for two patients in this study, additional cases can also further define what treatments might be effective for affected males carrying a *SYCP2* LOF variant, and the likelihood of success with those interventions. 

It is also important to note that the relationship between *SYCP2* and male infertility does not extend to female infertility. The maternal inheritance of a *SYCP2* LOF variant has been observed in two families, including patient 2 from this study, suggesting that female carriers can be fertile (Table 1) [8]. In addition, *Sycp2*-mediated pathogenicity has been shown to cause male infertility but only female subfertility in a mouse model [12]. It has been proposed that the *SYCP2* homologue *SYCP2L* may play an important role in female fertility instead, as has been shown in mice and humans [35,36].

The new cases presented in this study are instrumental in upgrading the clinical validity of the *SYCP2* and male infertility gene–disease association from on the border of limited and moderate to strong, supporting a causal relationship between the heterozygous LOF variants in *SYCP2* and autosomal dominant male infertility. This study shows the value of aggregating case reports to advance knowledge regarding genetic diseases and the importance of re-evaluating gene–disease validity classifications as new evidence arises. The impacts on gene panel design and variant interpretation resulting from this study can improve the diagnosis, genetic counseling, and therapeutic intervention for affected individuals. 

## Figures and Tables

**Table 1:** Cases with heterozygous loss-of-function variants in *SYCP2* and a low sperm count.

Individual	Age at Diagnosis	Variant in *SYCP2*	Exon	Clinical Diagnosis	Inheritance	Reference
M1686	29	NM_014258.4:c.2022_2025del p.(Lys674Asnfs*8)	24	cryptozoospermia	Unknown	[8]
M1581	27	NM_014258.4:c.2793_2797del p.(Lys932Serfs*3)	31	cryptozoospermia	Maternal	[8]
M1401	39	NM_014258.4:c.3067_3071del p.(Lys1023Leufs*2)	33	azoospermia	Unknown	[8]
Patient 1	30	NM_014258.4:c.89dup p.(Leu30Phefs*4)	4	oligoasthenozoospermia	*De novo*	This study
Patient 2	35	NM_014258.4:c.946_947del p.(Ser316Ilefs*7)	14	oligoasthenozoospermia	Maternal	This study
Patient 3	47	NM_014258.4:c.4378_4379del p.(Arg1460Alafs*10)	42	severe	Unknown	This study
				oligoasthenozoospermia		

**Table 2:** Genetic evidence summary matrix for evaluating the strength of the gene–disease association between *SYCP2* and autosomal dominant male infertility. Evidence for this gene–disease association was curated using the Clinical Genome Resource (ClinGen) framework, version 9 [6].

Genetic Evidence: Case-Level Data					
Evidence Type	Case Information	Suggested Point Upgrades		Points Given	References/Notes
		Functional Data	*De Novo*		
Variant Evidence: Autosomal Dominant	Predicted or proven null variant (default 1.5 points, scoring range 0–3 points per variant)	+0.5	+0.5	9.5	Six frameshift variants in *SYCP2* resulting in premature termination codons have been found in men with infertility (see Table 1 with cases from this study and [8]). All the variants are predicted to cause nonsense-mediated decay of the transcripts as the premature termination codons reside greater than 50 bp upstream of the last exon–exon junction [20]. All cases were assigned the default of 1.5 points aside from patient 1 from this study, who received an additional 0.5 points due to the variant occurring *de novo*.
	Other variant type with some evidence of gene impact (default 0.1 points, scoring range 0–1.5 points per variant)	+0.4	+0.4	0	No evidence available
Segregation Evidence	Evidence of segregation in one or more families (scoring range 0–3 points)			0	No evidence available
**Genetic Evidence: Case-Control Data**					
**Case-Control** **Study Type**	**Case-Control** **Quality Criteria**	**Suggested Points/Study**		**Points** **Given**	**References/Notes**
Single Variant Analysis	Variant detection methodology Power Bias and confounding factors Statistical significance	0–6		0	No evidence available
Aggregate Variant Analysis		0–6		0	No evidence available
**Total Genetic Evidence Points** **(out of 12 Total Allowable Points)**				9.5	

**Table 3:** Experimental evidence summary matrix for evaluating the strength of the gene–disease association between *SYCP2* and male infertility. Clinical validity classification of this gene–disease association was curated using the Clinical Genome Resource (ClinGen) framework, version 9 [6].

Experimental Evidence					
Evidence Category	Evidence Type	Suggested Points		Points Given	References/Notes
		Default	Range		
Function	Biochemical function	0.5	0–2	0.5	*SYCP2* encodes synaptonemal complex protein 2, a component of the lateral element substructure of the synaptonemal complex (SC) [9,10]. The SC is essential for the meiotic process of synapsis, which is a critical step in spermatogenesis [22]. In *Saccharomyces cerevisiae*, mutant *red1*, the yeast functional homolog of *SYCP2*, fails to assemble the SC during meiotic prophase [24].
	Protein interaction	0.5	0–2	0.5	The coiled-coil domain of mouse SYCP2 interacts with SYCP3 and TEX11, which have both been implicated in male infertility in humans [12,26,27,28].
	Expression	0.5	0–2	0.5	*SYCP2* is expressed predominantly in testis tissue, where spermatogenesis occurs [23].
Functional Alteration	Patient cells	1	0–2	0.5	Testicular biopsy histopathology from an individual with a heterozygous loss-of-function (LOF) variant in *SYCP2* (participant M1401) shows a phenotype of meiotic arrest at the pachytene spermatocyte stage [8].
	Non-patient cells	0.5	0–1	0	No evidence available
Models	Non-human model organism	2	0–4	4	Homozygous coiled-coil domain-deficient *Sycp2* mice demonstrate male infertility and meiotic arrest (2 points) [12]. Testes from *sycp2*^−/−^ zebrafish lack both spermatids and spermatozoa, resulting in the inability to fertilize eggs following natural mating with wild-type fish (2 points) [25].
	Cell culture model	1	0–2	0	No evidence available
Rescue	Rescue in human	2	0–4	0	No evidence available
	Rescue in non-human model organism	2	0–4	0	No evidence available
	Rescue in cell culture model	1	0–2	0	No evidence available
	Rescue in patient cells	1	0–2	0	No evidence available
**Total Experimental Evidence Points** **(out of 6 Total Allowable Points)**				6	

## Data Availability

All data generated or analyzed in this study are available upon request.
